# Role of intravenous immunoglobulin therapy in the survival rate of pediatric patients with acute myocarditis: A systematic review and meta-analysis

**DOI:** 10.1038/s41598-019-46888-0

**Published:** 2019-07-18

**Authors:** Chun-Yu Yen, Miao-Chiu Hung, Ying-Chi Wong, Chia-Yuan Chang, Chou-Cheng Lai, Keh-Gong Wu

**Affiliations:** 1 Department of Pediatrics, Taipei Veterans General Hospital and National Yang-Ming University, Taipei, Taiwan Republic of China; 2 Division of Infectious Diseases, Department of Pediatrics, Taipei Veterans General Hospital and National Yang-Ming University, Taipei, Taiwan Republic of China

**Keywords:** Cardiology, Cardiomyopathies, Viral infection

## Abstract

The treatment of pediatric myocarditis is controversial, and the benefits of intravenous immunoglobulin (IVIG) are inconclusive due to limited data. We searched studies from PubMed, MEDLINE, Embase, and Cochrane Library databases since establishment until October 1st, 2018. Thirteen studies met the inclusion criteria. We included a total of 812 patients with IVIG treatment and 592 patients without IVIG treatment. The meta-analysis showed that the survival rate in the IVIG group was higher than that in the non-IVIG group (odds ratio = 2.133, 95% confidence interval (CI): 1.32–3.43, p = 0.002). There was moderate statistical heterogeneity among the included studies (I^2^ = 35%, p = 0.102). However, after adjustment using Duval and Tweedie’s trim and fill method, the point estimate of the overall effect size was 1.40 (95% CI 0.83, 2.35), which became insignificant. Moreover, the meta-regression revealed that age (coefficient = −0.191, 95% CI (−0.398, 0.015), p = 0.069) and gender (coefficient = 0.347, 95% CI (−7.586, 8.279), p = 0.93) were not significantly related to the survival rate. This meta-analysis showed that IVIG treatment was not associated with better survival. The use of IVIG therapy in acute myocarditis in children cannot be routinely recommended based on current evidence. Further prospective and randomized controlled studies are needed to elucidate the effects of IVIG treatment.

## Introduction

Myocarditis is defined as inflammation of the myocardium with variable clinical presentation ranging from subclinical disease to heart failure, arrhythmia, fulminant hemodynamic collapse, and mortality^1^. Although myocarditis and idiopathic dilated cardiomyopathy (DCM) are considered distinct diseases, myocarditis frequently presents with a phenotype of new-onset DCM^2^. The predicted annual incidence of myocarditis is 1 to 2 cases per 100,000 children^3^.

Pediatric patients with myocarditis are stratified into 40 to 50% with the acute type and 30 to 40% with the fulminant type^4^. Acute myocarditis is defined as presenting with a less distinct onset of illness, established ventricular systolic dysfunction, and possible progression to DCM. Although the outcome of acute myocarditis is favorable in about 50% of cases, sequelae and chronic evolution occur in about 20% of cases, with 80% of cases of chronic cardiomyopathy leading to heart transplantation or death^5^. On the other hand, a subset of patients develop fulminant myocarditis (FM) presenting with severe cardiovascular compromise within two weeks since the onset of symptoms after a distinct viral infection prodrome^6^. Despite the severity of illness, most patients with FM regain native ventricular function if the cardiorespiratory and end-organ functions can be adequately supported until myocardial recovery. The survival rate of FM is around 51.6–80% which indicates the importance of prompt adoption of mechanical circulatory support to prevent rapid clinical deterioration and to reduce mortality rate^7–9^.

The majority of children with myocarditis present with an acute or fulminant disease, and infectious etiologies, particularly viral, are most common. Ventricular systolic dysfunction often normalize in patients surviving the acute illness^6^. Damage to the myocardium in acute myocarditis may be mediated by predominantly immunological mechanisms rather than by the direct effect of viral infection and replication^10^. High-dose intravenous immunoglobulin (IVIG) has shown potential in the treatment of myocarditis, hypothetically due to its antiviral, antibacterial, and immunosuppressant properties^11^. In one randomized multi-center trial, 41 adults (age 19–80 years) had improved survival with IVIG treatment^12^. However, another randomized controlled study of adults reported that IVIG did not improve the left ventricular ejection fraction (LVEF) or event-free survival^13^.

The treatment of pediatric myocarditis remains controversial, and the benefits of IVIG are inconclusive due to limited data^14^. Multivariable analysis in Pediatric Cardiomyopathy Registry (PCMR) study found no association of IVIG or corticosteroids with survival nor left ventricle normalization^15^. However, several studies have shown that IVIG treatment in children can be effective in improving LVEF^16–18^ and is beneficial for survival in children^19–21^.

Despite these discrepancies, IVIG is frequently used in current practice to treat acute myocarditis in adult and pediatric populations. Ghelani *et al*. conducted a multi-institutional study in the United States and found more than 70% of the pediatric patients were treated with IVIG^5^. Therefore, we performed this evidence-based meta-analysis and systematic literature review of survival outcomes of children with acute myocarditis after IVIG treatment.

## Methods

### Data sources and search strategy

We identified studies from PubMed, MEDLINE, Embase, and Cochrane Library databases since establishment until October 1st, 2018 (the date of the last literature search). All articles included in the present study involved human clinical studies published in English. The search parameters included the terms “myocarditis or cardiomyopathy” combined with “IVIG or immunoglobulin” and “children or pediatric”. This meta-analysis was performed in accordance with the Preferred Reporting Items for Systematic Reviews and Meta-analyses (http://www.prisma-statement.org/).

### Eligibility criteria and study selection

**Figure 1:**
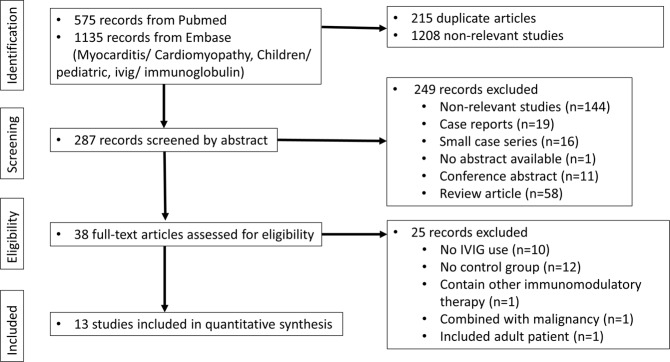
Selection of studies included in the analysis.

**Figure 2:**
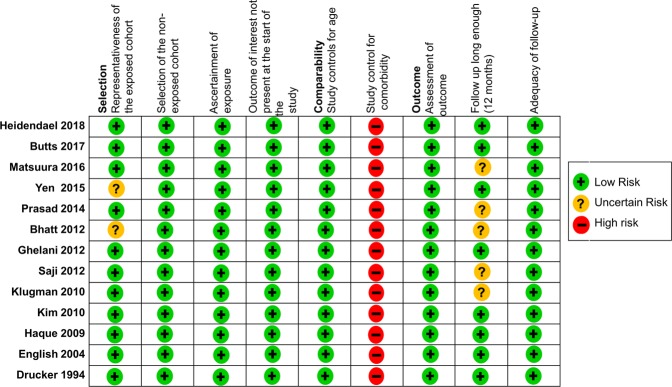
Risk of bias assessments for cohort studies.

We initially focused the literature search on randomized controlled trials (RCTs), and prospective and retrospective cohort studies. With the broad keyword search, 1710 studies were found. After a detailed inspection, 215 duplicates and 1208 non-relevant studies were found. Non-relevant studies, case reports, case series, conference abstracts, and review articles were all excluded. We also excluded studies in which the participants (1) were older than 18 years of age; (2) had received other immunomodulatory therapy; (3) had concurrent malignancies, and (4) were reported to have chronic myocarditis (Fig. 1). Finally, we only identified one quasi-randomized study and twelve retrospective cohort studies that were eligible. The risk of bias assessments of the included articles was rated using an adapted version of the Newcastle-Ottawa Scale for cohort studies, with a maximum score of 9 points (Fig. 2).

### Outcomes

The primary outcome of the present study was the survival rate following IVIG therapy. The endpoint of survival rate differed in each study from the time of discharge to 15 years. The secondary outcome was the recovery of ventricular function. However, five studies did not provide related information. The measurements of ventricular function were different in the studies (mean time to recovery, LVEF recovery percentage, LVEF recovery rate, etc.). Therefore, we only analyzed the primary outcome in our study.

### Data extraction

Data were independently extracted by two authors (Dr. Yen and Dr. Wu). Any disagreement was resolved by consensus. Data on the following measures were extracted: study design, diagnosis, sample size, treatment regimen, and authors’ conclusions (Table 1). We also extracted the mean age, gender, disease duration, supportive therapy, initial heart condition, statistical results, and survival rate from the enrolled studies (Table 2). There was no available adjusted odds ratio for the survival rate of IVIG treatment provided in the included studies.

**Table 1:** Summary of the included studies.

Study (year)	Study design	Diagnosis	Sample size (IVIG/Non-IVIG)	IVIG regimen	Author’s conclusion
Heidendael, Den Boer *et al*.^17^	Retrospective cohort study	Biopsy-proven or clinically diagnosed viral myocarditis or dilated cardiomyopathy due to viral infection	94 children: 21/73	2 g/kg	New onset dilated cardiomyopathy (either viral or idiopathic origin) ◎ Did not influence transplant-free survival ◎ Better improvement in LVEF ◎ Better recovery
Butts, Boyle *et al*.^26^	Retrospective cohort study	Newly confirmed myocarditis and clinically diagnosed myocarditis	55 children: 44/11	No dosing data	◎ Not associated with mortality ◎ Not associated with heart transplantation, shortening fraction at discharge
Matsuura, Ichida *et al*.^4^	Nationwide survey	Biopsy-proven in 19.2% of cases Acute myocarditis (65.6%), Fulminant myocarditis (33.5%)	237 children: 142/75	No dosing data	◎ Not affect the survival in the whole study population ◎ Better survival in fulminant myocarditis subgroup
Yen, Huang *et al*.^19^	Retrospective cohort study	Culture-confirmed enterovirus infection, Clinical evident myocarditis	15 neonate: 7/8	2–2.5 g/kg	In defined severe neonatal enterovirus infections ◎ Beneficial for survival
Prasad and Chaudhary^18^	Retrospective cohort study	Clinically diagnosed acute myocarditis	28 children: 12/16	1 g/kg/day (for 2 days)	◎ Beneficial for survival ◎ Improved recovery of LVEF ◎ Reduction in the episodes of fulminant arrhythmias
Bhatt, Sankar *et al*.^20^	Quasi-randomized control study	Acute encephalitis syndrome complicated by clinically diagnosed myocarditis	83 children: 26/57	400 mg/kg/day (for 5 days)	Children with AES complicated by myocarditis ◎ Beneficial for survival ◎ Improved recovery of LVEF
Ghelani, Spaeder *et al*.^5^	Retrospective cohort study	Biopsy-proven or MRI diagnosed acute myocarditis	514 children: 359/155	No dosing data	◎ No difference in transplant-free survival
Saji, Matsuura *et al*.^25^	Nationwide survey	1 Biopsy-proven in 33.1% of cases 2 Acute myocarditis (58%), Fulminant myocarditis (42%)	44 children: 29/15	1–2 g/kg/day (for 1–2 days)	◎ No difference in survival
Klugman, Berger *et al*.^27^	Retrospective cohort study	Clinically diagnosed myocarditis	216 children: 98/118	No dosing data	◎ No difference in survival
Kim, Yoo *et al*.^28^	Retrospective cohort study	Clinically diagnosed myocarditis	33 children: 23/10	2 g/kg	◎ No difference in recovery of LVEF ◎ No difference in survival
Haque, Bhatti *et al*.^21^	Retrospective cohort study	Clinically diagnosed myocarditis	25 children: 12/13	2 g/kg/day (for 1 day)	◎ No difference in recovery of LVEF ◎ Beneficial for survival
English, Janosky *et al*.^24^	Retrospective cohort study	Biopsy-proven or clinically diagnosed viral myocarditis	34 children: 18/16	16 patients: 2 g/kg patients: 1 g/kg	◎ No difference in time to recovery of normal LVEF ◎ No difference in survival
Drucker, Colan *et al*.^16^	Retrospective cohort study	Biopsy-proven or clinically diagnosed viral myocarditis	46 children: 21/25	2 g/kg/day (for 1 day)	◎ Improved recovery of LVEF ◎ Better survival

IVIG: intravenous immunoglobulin, LVEF: left ventricular ejection fraction.

**Table 2:** Summary of the clinical and statistical data of the included studies.

Study (year)	Mean age (IVIG/Non-IVIG)	Gender (Male%) (IVIG/Non-IVIG)	Disease duration	Initial heart condition (IVIG/Non-IVIG)	Supportive therapy	Statistical result (IVIG/Non-IVIG)	Survival rate (IVIG/Non-IVIG) (F/u duration)
Heidendael, Den Boer *et al*.^17^	10/18 months	48%/56%	Acute (43% < 1 weeks)	No difference in echocardiography & MRI	1. No difference in inotropic therapy, ICU admission, ECMO rate 2. Mechanical ventilation: 62%/36% (p = 0.03)	Recovery rate within 5 years: 70%:43% (p = 0.045)	Transplant-free survival (5 years): 90%:71% (p = 0.24)
Butts, Boyle *et al*.^26^	10.6/16.1 years	50%/27.3%	N/A	Shortening fraction: 17.8%:33.6% (p < 0.01)	Steroids, inotropes, mechanical circulatory support used	1. Length of admission: 16.5 days: 4 days (p < 0.01) 2. Transplantation: 18.2%:0% (p = 0.13)	95.5%:100% (1 year) (p = 0.47)
Matsuura, Ichida *et al*.^4^	6.5 ± 5.3 years	51%	Median: 3 days (range: 3 to 60 days)	N/A	Steroids used	N/A	78.9%:72% (At discharge) (p = NS)
Yen, Huang *et al*.^19^	Neonate	N/A	N/A	N/A	N/A	N/A	57%:12% (15 years) (p = 0.089)
Prasad and Chaudhary^18^	<12 years	58%/56%	Acute (<3 months)	LVEF: 35.3%:33.5% (p > 0.05)	N/A	1. LVEF 6 months post-treatment: 62.2%:43.3% (p < 0.01) 2. VT/VF recovered: 2/3: 1/3 (p < 0.01) 3. AV block recovered: 4/5: 1/3 (p < 0.01)	83%:56% (6 months) (p = 0.032)
Bhatt, Sankar *et al*.^20^	4.4/ 4.7 years	Male in majority	Mean 50 days (between encephalitis and myocarditis	LVEF: 32.8: 33.2% (p = 0.78)	N/A	LVEF at discharge: 49.5: 35.9 (p = 0.001)	96%:77% (At discharge) (p = 0.061)
Ghelani, Spaeder *et al*.^5^	9.2 ± 6.8 years	64%	Median: 7 days (interquartile: 3–19 days)	N/A	Steroids, inotropes, mechanical circulatory support used	N/A	88%:89% (p = 0.65)
Saji, Matsuura *et al*.^25^	1 month- 17 years	47%	N/A	N/A	Steroids, mechanical circulatory support used	N/A	86%:46% (p = 0.008)
Klugman, Berger *et al*.^27^	<18 years	N/A	N/A	N/A	Inotropes, mechanical circulatory support used	N/A	93%:90% (At discharge) (p = 0.38)
Kim, Yoo *et al*.^28^	41/60 months	52.1%/60%	Acute (<2 weeks)	N/A	No difference in inotropic therapy, the use of ACEI, or ventilator care	Mean time to recovery of function: 68 days: 33 days (p = 0.485)	86%:80% (1 year) (p = 0.607)
Haque, Bhatti *et al*.^21^	7.3/12 months	50%:46%	N/A	LVEF: 17.5%:22.5% (p = 0.17)	Inotropes: 3: 1.5 (p = 0.001)	Recovery of left ventricular function: 49%:46% (p = 0.13)	91%:53% (At discharge) (p = 0.04)
English, Janosky *et al*.^24^	85.1/34 months	44%:56%	Acute (<2 weeks)	Dilated left ventricle: 38%:56% (p = NS)	1. Inotropes: 14: 12 (p = NS) 2. Intubation: 8: 11 (p = NS) 3. LVAD/ECMO: 2: 4 (p = NS) 4. Steroid used in both group	Mean time to recovery of function: 2: 2.8 months (p = NS)	70%:75% (5 years) (p = 0.85)
Drucker, Colan *et al*.^16^	<2 years	47%:56%	Acute (<3 months)	Cardiac index: 3.1: 3.39 (p = NS)	1. Inotropes: 90%:52% (p < 0.01) 2. ACEI: 88%:53% (p = 0.02)	Recovery of LV function at 12 months: 100%:37% (p < 0.001)	84%:60% (1 year) (p = 0.069)

IVIG: intravenous immunoglobulin, MRI: Magnetic resonance imaging, ICU: intensive care unit, N/A: not available, LVEF: left ventricular ejection fraction, NS: not significant, LVAD: left ventricular assist device, ECMO: extracorporeal membrane oxygenation, VT/VF: ventricular tachycardia/fibrillation, AV: atrioventricular, ACEI: angiotensin-converting enzyme inhibitors.

### Data analysis

We performed a meta-analysis on all studies that provided survival rates. Sensitivity analysis was conducted for each study. Heterogeneity was tested using the I^2^ test, and a fixed-effects model was used if heterogeneity was lacking. Trim and fill with funnel-plot-based method was tested for publication bias. All of these analyses were performed using Comprehensive Meta-Analysis Software version 3 (Biostat Incorporated, Englewood, New Jersey). The quality of evidence and grading of the strength of recommendations was assessed using Grades of Recommendation, Assessment, Development and Evaluation (GRADE)^22^. The GRADE approach categorizes evidence into high, moderate, low or very low quality, taking into account the limitations of trial design, the risk of bias, inconsistency, indirectness, imprecision, and publication bias^23^.

## Results

### Search results and study characteristics

Of 575 records from PubMed and 1135 records from Embase, thirteen studies met the inclusion criteria, including one quasi-randomized study and twelve retrospective cohort studies (Fig. 1). A summary of the study characteristics is presented in Table 1. All studies met the diagnosis of myocarditis by clinical symptoms and echocardiographic evidence of new left-ventricular dysfunction, and six of the thirteen studies also performed endo-myocardial biopsies^4,5,16,17,24,25^. Only two studies from a nationwide survey by the Japanese Society of Pediatric Cardiology and Cardiac Surgery (JPCCS) reported the effect of IVIG between two clinical types of acute and fulminant myocarditis^4,25^. We enrolled a total of 812 patients who received IVIG treatment and 592 who did not. Nine studies used a high-dose IVIG regimen with a dosage of 2 g/kg for 1 day, 1 g/kg for 2 days, or 400 mg/kg/day for 5 days. Four studies did not provide information regarding the dosage^4,5,26,27^. Two patients in the study by English *et al*. received a single dose of 1 g/kg^24^. Five studies showed that the IVIG group had a better survival rate, and four studies showed that the IVIG group was associated with better recovery of cardiac function^16–18,20^.

A summary of the clinical and statistical data is presented in Table 2. The age of the patients in all of the studies were <18 years. There was no statistical difference in the initial heart function between the IVIG and non-IVIG group in six studies^16–18,20,21,24^, but the patients in the IVIG group had poorer initial heart function in the study by Butts *et al*. Three studies reported no difference between the IVIG and non-IVIG groups in the requirement of inotropic drugs use, and two studies reported that the IVIG group had a higher rate of requiring inotropic drugs^16,21^. Corticosteroid drugs were used in five studies and there was no further statistic data to compare the IVIG and control group^4,5,24–26^. The IVIG group had a higher rate of angiotensin-converting enzyme inhibitors (ACEI) use in Drucker *et al*. study^16^. Six studies reported the etiology of myocarditis. Coxsackie B virus was the most common pathogen in four studies, followed by adenovirus and cytomegalovirus^17,20,24,25,28^. Yen *et al*. only investigated myocarditis in neonates with severe enterovirus infections^19^.

### Statistical analysis results

**Figure 3:**
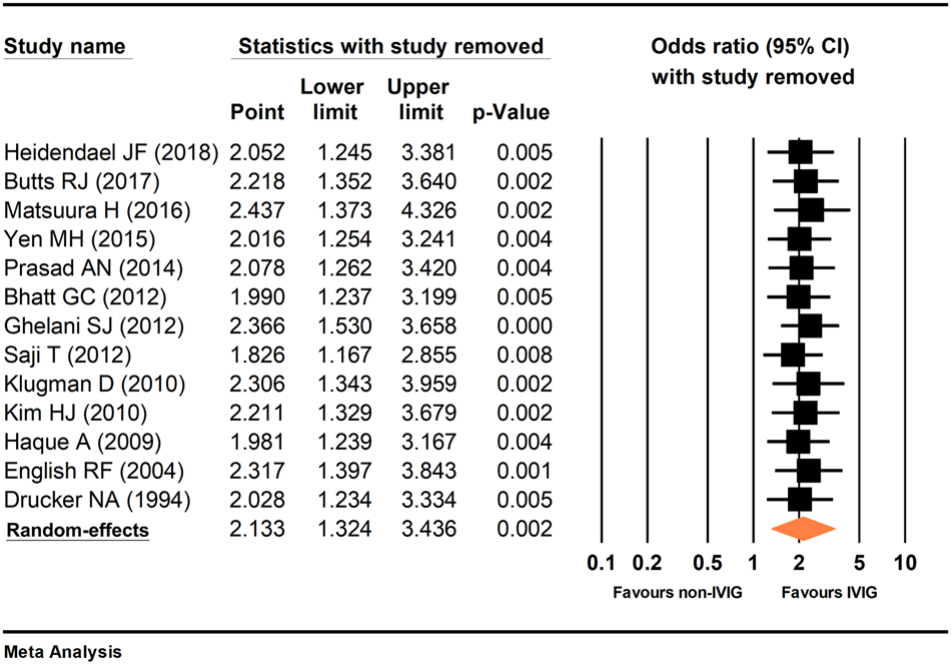
Sensitivity analysis. Forest plot showed each pooled result, having excluded a study, compared to the pooled result including all studies.

**Figure 4:**
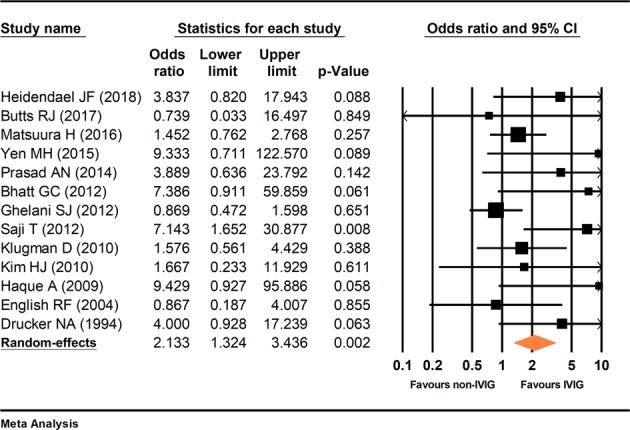
Forest plot. Comparison of survival rate between patients with IVIG and the control group.

The primary outcome of the current study was survival rate. The secondary outcome cannot be analyzed due to variations of the measurement method. The results of the sensitivity analysis are presented in Fig. 3. P-values remained significant throughout the leave-one-out sensitivity analysis. There was moderate statistical heterogeneity among included studies (I^2^ = 35%, p = 0.102). Additionally, each study had prominent heterogeneity including patient groups, disease etiology, the dosage of medication, and the tracking time, which may cause a substantial difference in the therapeutic result. Random-effects model was used. Compared with the control group, the patients who received IVIG treatment had a significantly higher survival rate (odds ratio = 2.133, 95% confidence interval (CI): 1.32, 3.43), p = 0.002) (Fig. 4). The meta-regression could only be performed in seven studies because age and gender were provided^4,5,17,21,24,26,28^. The meta-regression revealed that years of age (coefficient = −0.191, 95% CI (−0.398, 0.015), p = 0.069) and gender (coefficient = 0.347, 95% CI (−7.586, 8.279), p = 0.931) were not significantly related to the survival rate.

**Figure 5:**
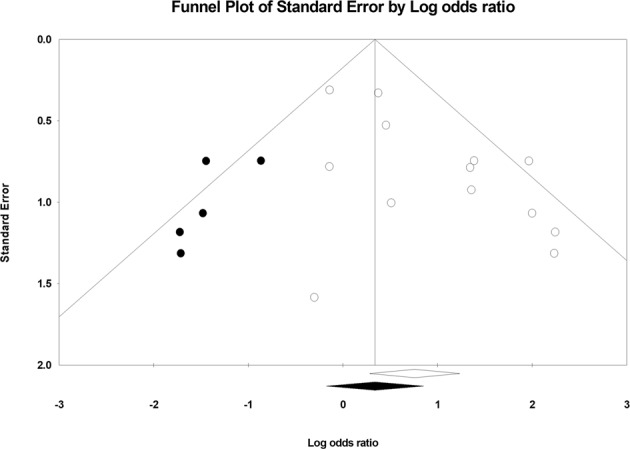
Funnel plot. Funnel plot for odds ratio of survival rate between patients with IVIG and the control group showed asymmetry, which meant publication bias. After running Duval and Tweedie’s trim-and-fill method by Comprehensive Meta-Analysis software, five missing studies were estimated.

The funnel plot (Fig. 5) for changes in the survival rate between the IVIG and non-IVIG group in 13 studies disclosed asymmetry (Egger test, p = 0.01 (2-tailed)) and visual funnel plot showed publication bias. In estimating the true effect size, the Duval and Tweedie’s trim-and-fill method was used. After running the algorithm using Comprehensive Meta-Analysis software, five missing studies were estimated. We found that the corrected point estimate of the overall effect size was 1.40 (95% CI 0.83, 2.35), which was statistically insignificant.

The quality of the evidence from the outcome evaluated by the GRADE system was assessed as low since the majority of included studies were retrospective cohort studies and the risk of bias was serious (Table 3). Hence, our confidence in the effect estimate is limited.

**Table 3:** GRADE assessment.

Study design	Risk of bias	Inconsistency	Indirectness	Imprecision	Publication bias	Large effect	Dose-response	Reduce all confounders	Quality	Importance
One quasi-randomized, twelve retrospective studies	Serious (−1)^†^	Low to Moderate^‡^	Not serious	Not serious	Detected (−1)	Nil^§^	Unknown	Nil	Low ⊕⊕○○^¶^	Critical

^†^Lack of randomization, blinding, allocation concealment, intention-to-treat analysis.

^‡^I^2^ = 35%, p = 0.102 (I^2^ < 40% may be low; 30–60% may be moderate; 50–90% may be substantial; 75–100% may be considerable).

^§^Odds ratio < 2 (95% CI 1.21, 2.36).

^¶^This research provides some indication of the likely effect. However, the likelihood that it will be substantially different (a large enough difference that it might have an effect on a decision) is high.

## Discussion

In this systematic review and meta-analysis, our result before trim and fill adjustment demonstrated that children who received IVIG treatment had a significantly higher survival rate than those who did not. Acute myocarditis can be caused by viral infections, but subsequent myocardial damage appears to be mediated by autoimmune mechanisms^29^. Previous studies have reported the therapeutic effects of high-dose IVIG in acute myocarditis^16,17,20^. The beneficial effect of IVIG leads to a more rapid clearance of myocardial viral infection via targeting specific antibodies to viruses^30^. IVIG can also modulate immune responses, which potentially result in decreased cardiac inflammation^31,32^. In patients with active lymphocytic myocarditis, those with circulating cardiac autoantibodies and no viral genome in the myocardium are the most likely to benefit from immunosuppression^33^.

IVIG has many immunomodulatory and anti-inflammatory effects at higher doses (2 g/kg over 2–5 days for adults or 2 days for children). Currently, more than 100 inflammatory and autoimmune disorders are treated with IVIG^34,35^. Eight studies used high-dose IVIG regimens (total of 2 g/kg) to treat acute myocarditis, including one study using 400 mg/kg/day for 5 days. The doses of the IVIG regimens were different in the enrolled studies and four studies did not report the dosage; thus subgroup meta-analysis could not be performed. Therefore, it is still unclear whether the duration of high dose IVIG affects the survival of patients with myocarditis.

Evidence from the Cochrane Collaboration Review of 2015, which was based on one trial, did not support the use of IVIG for the treatment of adults with presumed viral myocarditis due to lack of evidence of improved survival. In addition, the only pediatric trial included had a high risk of bias, but suggested that benefits may be seen in children with acute encephalitis syndrome (AES) complicated by myocarditis^36^. Bhatt *et al*. included patients aged 2 to 12 years with the mean age of 4.6 years^20^. The suspected pathogen was enterovirus, but definitive diagnosis could only be established in 14 children. Therefore, it is not clear whether the benefit of IVIG treatment extends to neonates or older children with myocarditis alone, as well as for other viral etiologies. It is possible that the contrasting effects of IVIG in children compared with adults may be due to the greater role of the immune response in myocarditis and enhanced viral etiology in children. It is also possible that children have a better potential for recovery of ventricular function and higher rates of survival^37^.

The variation in the efficacy of IVIG across different settings may be due to differences in the specific immunoglobulin components in each bottle, which is dependent on the protocols used in preparing IVIG^11^. Over 25 IVIG preparations are currently available worldwide, and each is derived from plasma pooled from at least 1000 donors. The characteristics of the various products may differ in immunoglobulin types, subclass distribution and antibody content, which may result in variable efficacy of IVIG^38,39^. In addition, the efficacy of IVIG may also be influenced by the heterogeneity of the disease etiology and the age of the patients, as most studies did not have details of the disease etiology. The onset of myocarditis is frequently triggered by viral infections caused by common respiratory viruses, enteroviruses and herpesviruses^40^. There is a growing list of viruses that may cause myocarditis with very few virus-targeted treatment options. Since most patients with acute myocarditis are diagnosed weeks after viral infection, it is relatively late to provide antiviral therapy upon the onset of myocarditis by when viral replication has often been cleared^41^. Furthermore, the confirmed diagnosis of viral etiology remains difficult to establish^17^.

The initial heart function may influence the survival outcome. In the study by Haque *et al*., even though the children in the IVIG group had a worse LVEF and increased need for inotropic drugs, the difference in survival rates between the groups persisted to be significant to demonstrate better outcomes in the IVIG group^21^. Similarly, the survival rate of the IVIG group in Drucker *et al*.’s study, in which the children also had increased usage of inotropic therapy, was significant as well^16^. However, in the study by Butts *et al*., the IVIG group did not have a better survival rate, and this finding may have been influenced by the poorer initial heart function in this group^26^. Data of initial heart condition was insufficient and not uniformly assessed.

Four studies revealed that the administration of IVIG was associated with significantly greater improvements in left ventricular function in the children^16–18,20^, although three studies reported conflicting results^24,26,28^. Nevertheless, different parameters of ventricular function were assessed in the studies (mean time to recovery, LVEF recovery percentage, LVEF recovery rate, etc.), and thus meta-analysis was not possible. The definite mechanism by which IVIG potentially improves myocardial dysfunction in myocarditis is still unknown. The anti-viral and anti-inflammatory effects of IVIG may play a role in improving myocardial dysfunction.

Fulminant myocarditis is characterized by acute hemodynamic collapse, fatal arrhythmias, or low cardiac output^42^. Interestingly, IVIG administration correlated with significantly better survival in patients with FM (59.6% vs. 15.0%, P < 0.005) than acute myocarditis (90% vs. 92.7%, P = not significant)^4^. The same result could be found in the study done by Saji *et al*.^25^. In patients with FM, the survival rate tended to be higher in those receiving either IVIG, steroids or mechanical support, in comparison to those who did not receive these treatments. Therefore, there is some argument that fulminant myocarditis should be analyzed separately. However, the other eleven studies did not provide information regarding a further clinical type of myocarditis. Therefore, subgroup meta-analysis comparing the acute myocarditis and FM cannot be analyzed.

Additionally, concomitant administration of medication can affect the survival rate between the study groups. It has been established in the PCMR analysis study that children with dilated cardiomyopathy have improved survival in the more recent era (1990 to 1999). This appears to be associated with the comprehensive treatment of inotropic support, mechanical ventilation, and mechanical circulatory support other than heart transplantation. Medication such as diuretics, beta-blockers and angiotensin-converting enzyme inhibitors may also play a role^9^. Several studies have demonstrated ACEI can slow progression of reverse remodeling and reduce mortality rates in heart failure patients^43^. In Drucker *et al*. study, the IVIG group had a higher rate of ACEI prescription. Therefore, the result of improved survival in this study may be associated with medications apart from IVIG. A review from the Cochrane Database found that corticosteroids treatment did not reduce mortality in myocarditis patients^44^; nevertheless, the role of corticosteroid in myocarditis remains controversial. Corticosteroids were administered in five included studies, though dosage details between the IVIG and control groups were not recorded^4,5,24–26^. The influence of corticosteroids on both groups was difficult to assess.

There were only seven studies providing detailed information of both age and gender^4,5,17,21,24,26,28^, which could be potentially important variables. We therefore examined the impact of age and gender on IVIG effect size by using the meta-regression models. As a result, gender was not associated with survival rate apparently (p = 0.93). Notably, the beneficial effect of IVIG on survival was more evident in infants and young children^19–21,25^, in which age group enterovirus infection is common^45^. However, the efficacy of IVIG use in neonatal enterovirus infection remains uncertain. Furthermore, the meta-regression result revealed that age was not significantly related to the survival rate (coefficient = −0.191, 95% CI (−0.398, 0.015), p = 0.069).

Compared with the previous study published in Cochrane Reviews which stated IVIG should not be provided as routine practice^36^, we added twelve retrospective cohort studies to the meta-analysis in order to strengthen the level of evidence. Since Egger test showed publication bias, we used Duval and Tweedie’s trim-and-fill method, a funnel plot-derived and two-step method, to estimate the number of unpublished counterparts and provide a summary effect adjusted for publication bias^46^. As a result of adjustment, IVIG treatment was not associated with better survival. Therefore, evidence for IVIG therapy in acute myocarditis remains limited.

### Study limitations and considerations

There are several limitations to this study. Firstly, only one RCT was available for analysis. Currently, no prospective randomized controlled trials in literature demonstrated the efficacy of IVIG in children with acute myocarditis. The key reason is that acute myocarditis is rare, with an estimated annual incidence of 0.26 per 1000,000 children from a nationwide survey by JPCCS^25^. Moreover, pediatric myocarditis is commonly associated with rapid progression to heart failure^47^, and thus raises the difficulty of conducting a large-scale, randomized trial of IVIG. Hence, the power of this study is limited by the availability of therapeutic information from a small population of patients without random assignment to treatments. However, we included a total of thirteen studies to improve the power of evidence.

Secondly, there was high inter-study variability between the included studies. The diagnostic criteria of myocarditis, the etiologies, IVIG regimens, and the combination of other treatment in these studies were different. We therefore performed heterogeneity testing and sensitivity analysis. The result of I^2^ test was 35% with p-value = 0.102, implying that the heterogeneity of the studies was moderate. The strength of pooled estimate did not significantly differ according to the characteristics of individual studies in the leave-one-out sensitivity analysis (Fig. 3). In consideration of moderate heterogeneity, the meta-analysis was performed using a random-effects model.

## Conclusion

In conclusion, this meta-analysis demonstrated that IVIG treatment for acute myocarditis in children was not associated with better survival rate. The quality of evidence is low and the strength of recommendation according to the GRADE system is weak. Taken together, the use of IVIG therapy in acute myocarditis in children cannot be routinely recommended based on current evidence. However, given the variability and limited number of the included studies, further prospective, randomized controlled studies are needed to elucidate the effects of IVIG treatment.
